# Cost minimization in breast conserving surgery: a comparative study of radiofrequency spectroscopy and full cavity shave margins

**DOI:** 10.1186/s12962-023-00477-1

**Published:** 2023-09-16

**Authors:** Richard Gilmore, Jennifer Chen, Robert Dembinski, Yannis Reissis, David Milek, Lisa Cadena, Mehran Habibi

**Affiliations:** 1 Director, Breast Program at Staten Island University Hospital, Chief of Breast Surgery, Western Region, Northwell Health, 256 Mason Ave., Building B, 2nd Fl., Staten Island, NY 10305 USA; 2 Department of Surgery, Northwell Health, Zucker School of Medicine, New York, United States

**Keywords:** MarginProbe, Breast-conserving surgery, Positive margins, Intraoperative assessment, Radiofrequency spectroscopy

## Abstract

**Background:**

In an effort to minimize positive margins and subsequent re-excision after breast conserving surgery (BCS), many providers and facilities have implemented either a Full Cavity Shave (FCS) approach or adding the MarginProbe Radiofrequency Spectroscopy System.

**Objective:**

We sought to create a functioning Pro-Forma for use by facilities and payers to evaluate and compare the cost savings of implementing FCS or MarginProbe based on personalized variable inputs.

**Methods:**

A decision tree demonstrating three possible surgical pathways, BCS, BCS + FCS, and BCS + MarginProbe was developed with clinical inputs for re-excision rate, mastectomy as 2nd surgery, rate of reconstruction, and rate of 3rd surgery derived by a literature review. A surgical pathway cost formula was created using the decision tree and financial inputs derived by utilizing the nation’s largest database of privately billed health insurance claims and Medicare claims data (fairhealth.org). Using the surgical pathway formula and financial inputs, a customizable Pro-Forma was created for immediate cost savings analysis of BCS + FCS and BCS + Marginprobe using variable inputs. Costs are from the perspective of third-party payers.

**Results:**

Utilizing MarginProbe to reduce re-excisions for positive margins can be associated with better cost-savings than FCS due to the increased pathology processing costs by using an FCS approach. The reduction in re-excision provided by both FCS and MarginProbe offset their increased expense to various degrees with cost savings of each method improving as baseline re-excisions rates increase, until ultimately each may become cost-neutral or cost-prohibitive when compared to BCS alone. Our data suggest that in the privately insured population, MarginProbe provides a cost-savings over BCS alone when baseline re-excision rates are over 20% and that FCS becomes cost-saving when baseline re-excision rates are over 29%. For Medicare patients, MarginProbe provides a cost-savings when baseline re-excision rates exceed 34%, and FCS becomes cost-saving for re-excision rates over 52%. Our Pro-Forma allows an individual provider or institution to evaluate the cost savings of the FCS approach and/or utilization of the MarginProbe device such that the additional cost or cost-savings of utilizing one or both of these methods can be quickly calculated based on their facility’s volume and baseline re-excision rate.

**Conclusions:**

Our data suggest that utilizing either an FCS approach or the MarginProbe radiofrequency spectroscopy system may be a cost-saving solution to reducing the rate of re-excisions depending on a facility or practice’s surgical volume and baseline re-excision rate. The degree to which each of these interventions provides an added cost or cost-savings to healthcare payers can be evaluated by utilizing the Pro-Forma outlined herein with customizable variable inputs.

## Introduction

The American Cancer Society estimates that in 2023, over 350,000 women will be diagnosed with either invasive breast cancer or DCIS [1]. The majority (60–70%) of these patients will undergo Breast Conserving Surgery (BCS) also known as lumpectomy or partial mastectomy, as a less invasive alternative to total mastectomy [2, 3]. The goal of BCS is complete removal of the cancerous tumor while maintaining the cosmetic appearance of the breast. A meta-analysis of more than 1,500,000 patients found that when combined with adjuvant radiation therapy, BCS provides better survival to mastectomy in patients with early stage breast cancer [4].

Pathologically involved margins after lumpectomy double the risk of breast cancer recurrence, [5, 6] and typically necessitate a repeat operation to remove additional tissue and residual disease. In 2014, the Society of Surgical Oncology (SSO) and the American Society of Clinical Oncology (ASCO) announced a consensus guideline defining positive margins for early-stage invasive breast cancer as “no tumor on ink” [5]. This guideline was followed in 2016 by a 2 mm margin standard for DCIS, presented in a consensus statement by the SSO, ASCO and the American Society for Radiation Oncology (ASTRO) [6]. Despite widespread adoption of the consensus guidelines, the need for re-excision following lumpectomy is common, averaging 20% nationwide, ranging from less than 10% to greater than 70% amongst surgeons and institutions [7]. A recent analysis of 291,000 Medicare claims reported an overall re-excision rate of 19%, with wide variability amongst surgeons from 0 to 91.7%; moreover, the rate of re-excisions was found to decrease from 22% before the aforementioned “no tumor on ink” guideline to 17.2% afterward.^8^

Although breast re-excision is standard procedure in the setting of positive surgical margins, the additional surgery poses several risks for the patient, including increased incidence of complications, greater psychological and emotional burden, a decline in cosmetic outcomes secondary to larger tissue volume removal, and delays initiating adjuvant therapy. In addition, after partial mastectomy with oncoplastic reconstruction, a mastectomy is required if margins are found to be positive on final surgical pathology. Re-operations also lead to increased healthcare spending, adding additional financial burden to the patient and the healthcare system as a whole.

In an effort to reduce the need for reoperation, Full Cavity Shave (FCS) has emerged as an accepted approach for mitigating positive margins at the time of initial surgery. Surgeons utilizing FCS systematically remove additional margins from all aspects of the lumpectomy cavity at the time of BCS. Some studies have shown that rates of positive margins are substantially lower when additional cavity shaves are removed during initial surgery, leading to a decrease in re-excisions by up to 50% or more [8–13].

Alternatively, surgeons may choose to utilize the MarginProbe Radiofrequency Spectroscopy System (Dilon Medical Technologies, Newport News) as an adjunctive tool for intraoperative margin assessment. MarginProbe utilizes radiofrequency spectroscopy to algorithmically analyze and detect cancerous tissue at the margins of excised lumpectomy specimens in real-time. By providing immediate feedback at the time of surgery, surgeons can take directed shavings, removing additional tissue from only the areas of concern. Like FCS, MarginProbe clinical trials report reduction in re-excision by 50% or more [14–21].

Although the clinical importance of minimizing the need for breast re-excision is clear, the cost savings in doing so is less understood. Few studies have directly compared the financial impact of re-excision after BCS or the cost savings of FCS, and to our knowledge none have performed a cost analysis of MarginProbe in comparison to BCS or BCS + FCS. We conducted a cost savings comparison of utilizing FCS or MarginProbe during BCS and examined the costs including the associated downstream resource utilization of each, presented as a customizable Pro-Forma for individual economic evaluation by third-party payers.

## Methods

### Cost perspective

For the purpose of this study, cost saving is reported from the perspective of third-party payers including commercial insurers and Medicare, as opposed to facility or patient perspective. The emotional and financial toll of reoperation on patients is known and acknowledged. Any additional cost to facility or payer due to reoperation or the prevention or reoperation may also impact the patient. Facilities must balance the clinical need to reduce re-excisions with the reality of adding additional procedural cost for a medical device without reimbursement, while conversely receiving payment for re-excision by third-party payers. Therefore, the least biased method to evaluate cost saving of methods to reduce re-excision will be from the perspective of the third-party payer [22, 23].

### Clinical inputs

We searched the PubMed MEDLINE database for BCS outcomes related to average (invasive and DCIS) re-excision rates in the United States, published since 2014 [5–24, 12, 13, 25–36]. We also searched reduction in re-excision as a result of utilizing FCS [12, 13, 36, 37] and reduction in re-excision as a result of utilizing MarginProbe [15–21, 38, 39] during the same timeframe. The ratio of reoperations as re-excision BCS vs. conversion to mastectomy was researched for both single and multiple reoperations after BCS [30, 40–48]. Further, we reviewed the literature for rate of mastectomy that is bilateral, as well as the average rate of implant reconstruction after mastectomy [49–51]. The American Society of Plastic Surgeons 2020 Plastic Surgery Statistics Report provided details on timing of reconstruction (immediate or delayed) [52]. All mastectomy data was limited to the United States to avoid bias as a result of different decision guidelines in other healthcare systems. Our own institutional data was analyzed to supplement these references.

**Fig. 1:**
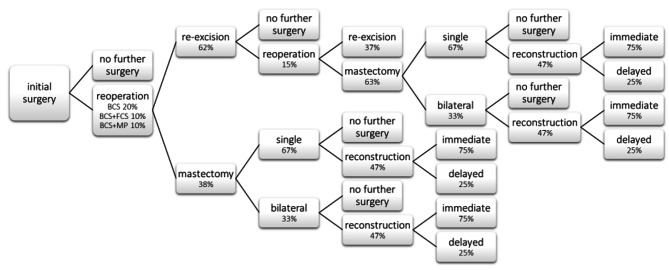
Surgical pathway decision tree. “MP” = MarginProbe

Using these clinical inputs, a decision tree was created illustrating the surgical care pathway for BCS alone, BCS + FCS or BCS + MarginProbe. (Fig. 1)

### Financial inputs

Fairhealth.org was used to gather commercial cost data. Medicare data was collected from CMS.gov. To complement this comparison, we gathered data on the number of people who have healthcare insurance from the most recent U.S. Census Bureau report on healthcare coverage in the United States [53] which included a breakdown of types of insurance. The CPT codes used for the purposes of this study are found in Table 1.

**Table 1:** CPT Codes used for cost savings comparison of FCS vs. MarginProbe Fairhealth.org does not provide national average costs, therefore 11 ZIP codes were chosen from 11 different states geographically covering all regions of the United States. The average cost between these states provided a surrogate for a national average cost. Cost variation by state was also calculated. The states and corresponding ZIP codes used are depicted in Table 2

CPT Code	Procedure
19,301	Mastectomy, partial (e.g., lumpectomy, tylectomy, quadrantectomy, segmentectomy)
19,303	Mastectomy, simple, complete
88,307	Surgical pathology, gross and microscopic examination
19,340	Insertion of breast implant on same day of mastectomy (i.e., immediate)
19,342	Insertion or replacement of breast implant on separate day from mastectomy

**Table 2:** States and ZIP codes comprising national average cost calculations

*ZIP Code*	*State*	*ZIP Code*	*State*	*ZIP Code*	*State*	*ZIP Code*	*State*
99,164	Washington	80,523	Colorado	55,959	Minnesota	10,001	New York
93,505	California	66,101	Kansas	49,254	Michigan	21,231	Maryland
59,634	Montana	77,590	Texas	32,313	Florida		

Although CPT codes for BCS and BCS + FCS are identical, pathology CPT code 88,307 is paid on each specimen analyzed. Therefore, a FCS operation with multiple specimens incurs additional cost for payers than a BCS with fewer specimens for pathology analysis.

MarginProbe costs were provided directly by the device manufacturer. A one-time cost for the MarginProbe is priced at $50,000. The list price for the disposable probe is $995 per procedure. Actual pricing for console and disposable probes may be lower based on IDN contract pricing and negotiated procedure volume discounts. All cost savings analyses use device list price unless stated otherwise.

### Price comparison formula

**Fig. 2:**
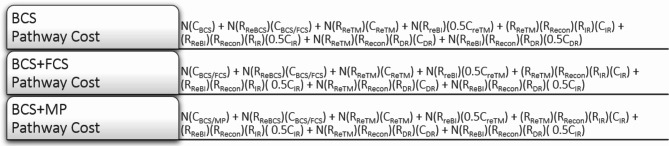
Surgical pathway cost formula. BCS, BCS + FCS, BCS + MP

Using the clinical and financial inputs in combination with the Decision Tree, a cost formula was created for each surgical pathway (Fig. 2) for input into a Pro-Forma Model.

**Table:** 

N = Number of Patients	BCS = Breast Conserving Surgery	Recon = Reconstruction
**R** = Rate	**FCS** = Full Cavity Shave	**IR** = Immediate Reconstruction
**C** = Cost	**MP** = MarginProbe	**DR** = Delayed Reconstruction
**Re** = Reoperation	**TM** = Total Mastectomy	**BI** = Bilateral Mastectomy

### Cost savings analysis

We referenced the Cost Savings Economic Evaluation framework provided by the Office of the Associate Director for Policy and Strategy on the cdc.gov website [54] to perform three analyses for output on the Pro-Forma: (1) The cost savings of adding FCS to BCS, (2) the cost savings of adding MarginProbe to BCS, and (3) the cost savings of MarginProbe and FCS compared to each other. In this case, where both MarginProbe and FCS reduce re-excisions and therefore cost to the payer, the two methods demonstrate different cost savings due to cost of preventing each positive margin.

Re-excision rate reduction as a result of implementing either MarginProbe or FCS to BCS alone were set by default to 50%. The rate of reduction can be adjusted in the Pro-Forma. The MarginProbe console and disposable probe costs are set at list price by default but can be adjusted in the Pro-Forma.

Net costs/net savings were calculated by subtracting the formula-calculated pathway cost from the pathway cost of its comparator. i.e., the net cost of adding FCS to BCS is the pathway cost of FCS minus the pathway cost of BCS alone. Net costs/net savings are presented on both a per patient basis and total annual basis based on BCS volume inputs in the Pro-Forma. When the intervention improves re-excision rates but is more costly, the cost savings is also presented as a ratio, reported as “cost per re-excision prevented.”

## Results

### Overview

All surgical pathway costs are dependent on multiple factors, including the patient’s insurance coverage, the provider’s current rate of re-excision, and the final rate of re-excision after adding FCS or MarginProbe to BCS. BCS + FCS pathway costs begin higher than BCS alone due to pathology charges for each of the additional shave specimens from the six anatomical faces of the lumpectomy cavity. BCS + MP costs begin higher than BCS alone due to an average two additional shave margins and device cost. The reduction in re-excision provided by adding FCS or MarginProbe to BCS may offset the increased spending to various degrees based on baseline re-excision rate, until ultimately, each may become cost-neutral or provide a cost-savings.

**Fig. 3:**
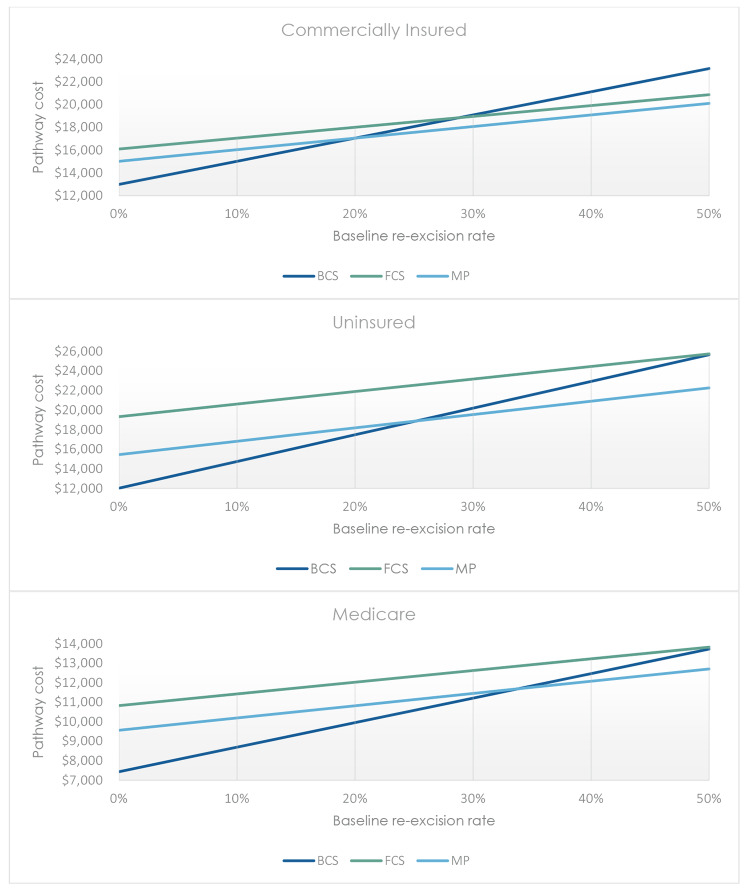
Associated pathway costs for BCS, BCS + FCS and BCS + MP across spectrum of baseline re-excision rates

In the commercially insured population, MarginProbe provides added cost to standard of care BCS when re-excision rates are below 20%, where it then becomes cost-neutral. Using an FCS approach adds additional cost when re-excision rates are below 29%, where it then becomes cost-neutral. For uninsured patients, MarginProbe becomes cost-neutral for a baseline re-excision rate of 25%, and FCS is cost-neutral when re-excisions reach 51%. Finally, in the Medicare population, MarginProbe was found to add additional cost up to a baseline re-excision rate of 34% where it becomes cost-neutral, and FCS provided added cost until a re-excision rate of 52% was reached. Pathway cost comparisons along the range of baseline re-excision rates in Commercially Insured, Uninsured, and Medicare patients are illustrated in Fig. 3.

### Using the pro-forma for customized analysis

Individualized analysis for consideration of surgical pathways across various patient populations, procedure volumes and current standard of care is made possible by utilizing the Pro-Forma. Examples A-B demonstrate its utility for evaluating the economic impact of adopting either method of margin management in additional to BCS, as well as comparing the effect of changing from one method to the other when it is already part of the facility standard of care.

### Example A – low volume facility, currently using BCS (can update examples however the group wants)

In this example of a typical 400 bed facility, inputs include an annual procedure volume of 45 lumpectomies of which 42% are Medicare and 58% are commercially insured. The re-excision rate is 19.6% and the analysis considers MarginProbe at list price.

Implementing FCS would add an additional $1,458 per patient. It would cost $14,881 to prevent one re-excision. Alternatively, adding MarginProbe to BCS procedures would incur an initial capital expenditure of $50,000 and an additional $397 per patient preventing one re-excision for every $4,055 spent.

**Fig. 4:**
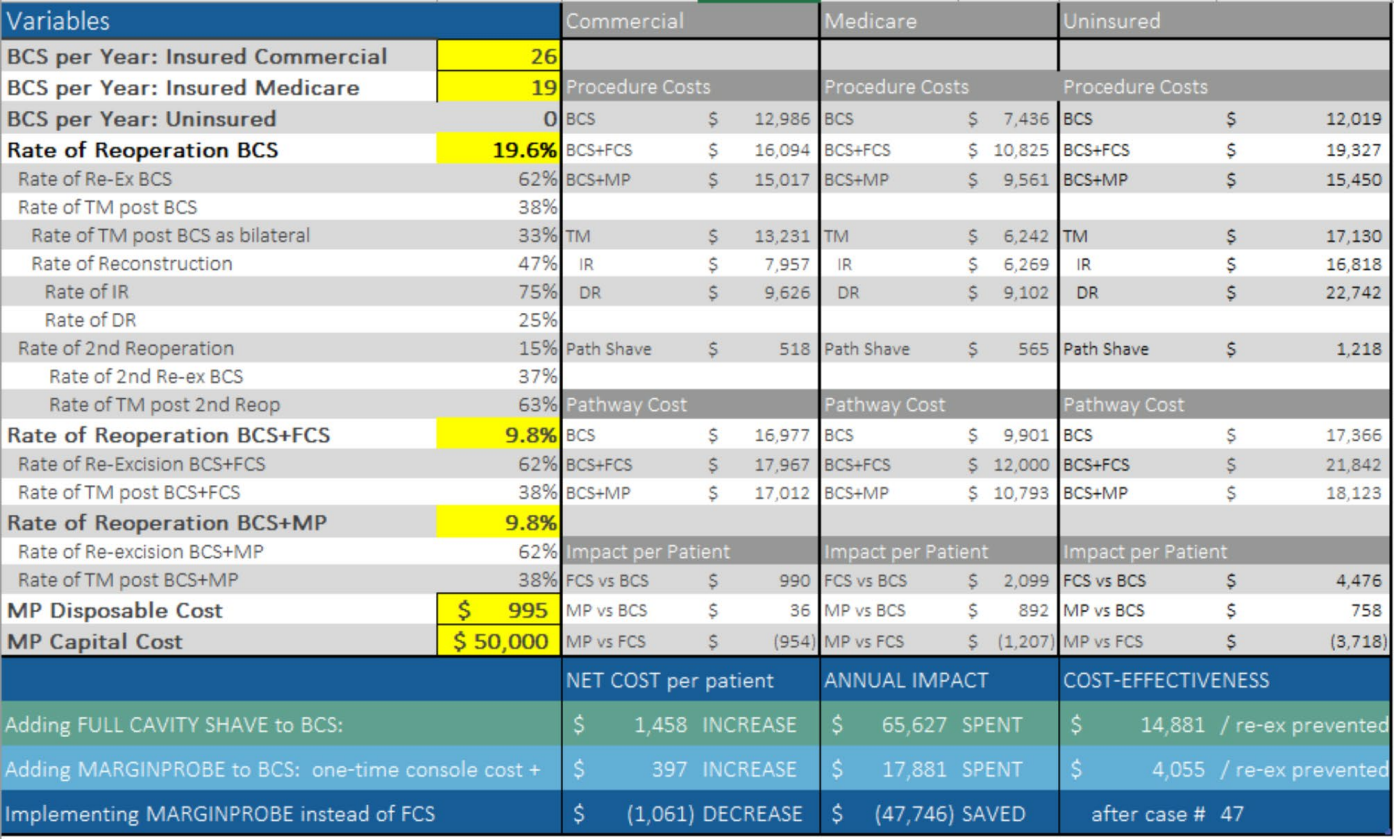
Pro-Forma example A

Comparing the two interventions directly, implementing a MarginProbe program in lieu of FCS would be cost-neutral for the first year due to the initial capital cost of the MarginProbe console, and then would reduce healthcare spend by nearly $1,061 per patient and save nearly $48,000 per year while reducing re-excisions below 10%. (Fig. 4)

### Example B – high volume facility, currently using FCS

This example represents a high-volume facility performing 400 BCS per year. FCS is standard of care for each procedure, and the re-excision rate is 5.1%. The payer-mix is 31% Medicare, 69% private insurance. MarginProbe variables are set to list price of $995 per disposable probe and a one-time cost of $50,000 for the system console.

**Fig. 5:**
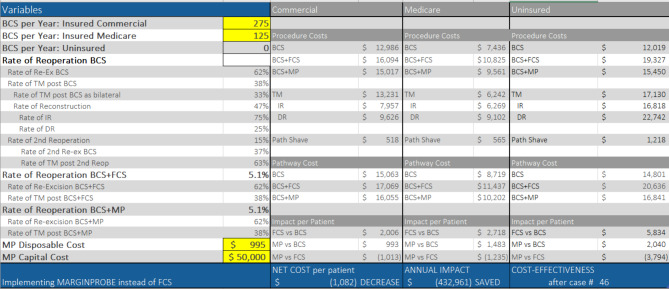
Pro-Forma example B

Converting to MarginProbe from FCS would reduce per patient cost by $1,082 and provide an annual healthcare savings of over $430,000 beginning after six weeks, while maintaining the same 5.1% re-excision rate. (Fig. 5)

## Discussion

Successful BCS is predicated on achieving pathologically clear margins. Reoperation due to margin involvement has been described as “the other breast-cancer epidemic” [55] and much focus has been placed on reducing re-excision rates across the United States.

Multiple best practices and methods have been accepted as part of a surgeon’s “toolbox” and meant be used simultaneously to minimize the need for re-excision surgery as recommended by the American Society of Breast Surgeons (ASBrS) [7]. Examples include localization of non-palpable lesions, specimen orientation of three or more margins, oncoplastic technique, and specimen radiograph with surgeon intraoperative review.

FCS has also been recommended as a part of the toolbox, as a method to reduce the burden of positive margins [7]. Cost-savings associated with FCS have been mixed [56–58]. Our Pro-Forma illustrates the dependency of baseline re-excision rate on the economic utility of FCS. Likewise, the cost of utilizing MarginProbe for intraoperative margin assessment may or may not be offset by reduction in re-excision, dependent on the facility’s beginning rate of re-excision.

Our Pro-Forma considers only the surgical phase of breast-cancer treatment, as the post-surgical treatment pathways would be the same for both FCS and MarginProbe patients. One shortcoming of the Pro-Forma is that it does not take into consideration radiation therapy costs, which does not impact a comparison of FCS and MarginProbe to each other but may alter the significance of the two methods in comparison to BCS alone, due to more conversion to mastectomy (and therefore less radiation) in a percentage of patients with positive margins undergoing standard BCS. It would not be difficult to adjust the Pro-Forma to consider radiation therapy costs based on a facility’s unique parameters.

There are no known head-to-head studies of FCS versus MarginProbe. Therefore, although the Pro-Forma allows for variable inputs for the rate of re-excision reduction of each, the assumption of each reducing reoperations by 50% is based on published literature of each method and is a current limitation of the Pro-Forma cost savings example reported.

Another way the Pro-Forma could be used is in only a subset of BCS patients at higher risk of positive margins and re-excision. For example, a facility utilizing intraoperative ultrasound for margin assessment during their 300 annual lumpectomies, with a re-excision rate of 4%, would see that although FCS and MarginProbe would lower re-excisions to 2%, it would be at an annual additional cost of nearly $850,000 for FCS or just over $500,000 for MarginProbe.

Conversely, the same facility could determine that their 12 re-excisions were nearly exclusively in DCIS patients and use the Pro-Forma to determine that utilizing FCS or MarginProbe only for their 36 annual DCIS patients per year, they would reduce their re-excisions by half, while saving $3,000 per year if using FCS, or saving almost $37,000 per year if using MarginProbe.

The personalization of the Pro-Forma enables exacting review and consideration to the cost and utility of the two surgical pathways. Any parameter can be attributed to a facility’s personalized input.

## Conclusions

The clinical importance of minimizing positive margins and subsequent re-excision after BCS is well known. MarginProbe Radiofrequency Spectroscopy is solution than reduce the positive margins and re-excisions by 50% or more. The degree to which each of these interventions provides an added cost or savings to healthcare payers is dependent on facility breast-conserving procedural volume and baseline re-excision rate. Comparison of the two interventions as well as overall cost savings to healthcare payers can be evaluated by utilizing the Pro-Forma with customized variable inputs. https://www.cancer.org/research/cancer-facts-statistics/all-cancer-facts-figures/2023-cancer-facts-figures.html

## Data Availability

The data and material used for this manuscript are publicly available and presented in the reference section.
